# Screening and Risk Analysis of Atrial Fibrillation After Radiotherapy for Breast Cancer: Protocol for the Cross-Sectional Cohort Study “Watch Your Heart (WATCH)”

**DOI:** 10.2196/67875

**Published:** 2025-06-04

**Authors:** Laura Saint-Lary, Baptiste Pinel, Loic Panh, Gaelle Jimenez, Julien Geffrelot, Youlia Kirova, Jeremy Camilleri, David Broggio, Marie-Odile Bernier, Corinne Mandin, Christelle Levy, Serge Boveda, Juliette Thariat, Sophie Jacob

**Affiliations:** 1 Laboratory of Epidemiology Autorité de Sûreté Nucléaire et Radioprotection Fontenay-aux-Roses France; 2 Department of Radiation Oncology Clinique Pasteur Toulouse France; 3 Department Heart Rhythm Management, Cardiology Clinique Pasteur Toulouse France; 4 Department of Radiation Oncology Centre François Baclesse Caen France; 5 Department of Radiation Oncology Institut Curie Paris France; 6 Department of Dosimetry Autorité de sûreté nucléaire et de radioprotection Fontenay-aux-Roses France

**Keywords:** breast cancer, radiotherapy, atrial fibrillation, smartwatch, arrhythmia, cardiac dosimetry

## Abstract

**Background:**

Atrial fibrillation (AF) after radiotherapy (RT) in patients with breast cancer (BC) is a relatively new and understudied topic. AF can increase the risk of stroke and other serious cardiovascular complications, compromising patients’ quality of life and survival. Screening of AF, both asymptomatic and symptomatic forms, is therefore essential for optimal management.

**Objective:**

The aim of the Watch Your Heart After Radiotherapy for Breast Cancer (WATCH) study is to assess the incidence of AF (symptomatic or asymptomatic) occurring throughout a 5-year follow-up after RT and to investigate whether cardiac radiation exposure is associated with the occurrence of such events.

**Methods:**

WATCH is a cohort study that will include 200 patients over 65 years old, treated with RT for BC 5 years before inclusion and without a history of AF. Cross-sectional screening for AF at the time of the scheduled 5-year post-RT visit will be conducted by recording data from a Withings ScanWatch smartwatch for 1 month, confirmed by an electrocardiogram (ECG), and validated by a physician. In addition, a transthoracic echocardiography (TTE) will be performed, providing a comprehensive assessment of cardiac structures, and allowing us to investigate the underlying etiology and assess the risk of complications. Patients’ medical records will provide retrospective information about the timing and risk factors for the occurrence of AF and other arrhythmias and cardiac diseases during the 5 years following RT. The development of deep learning algorithms for autosegmentation analysis of potentially critical substructures for the occurrence of AF, including cardiac chambers, the sinoatrial node, the atrioventricular node, coronary arteries, and pulmonary veins (PVs), will produce dosimetry linked to previous RT treatment for all contoured structures.

**Results:**

Enrollment started in October 2023 and will continue until mid-2026 to include 200 patients, which will ensure an 80% statistical power to detect a significant difference in AF incidence around 15% for the group of patients moderately exposed (<75th percentile of the mean heart radiation dose) and 25% for the group of patients highly exposed (>75th percentile of the mean heart radiation dose). The results are expected by the end of 2026.

**Conclusions:**

This study will contribute to generating new knowledge on AF after RT for BC and help considering the inclusion of AF screening into routine clinical practice for these patients. Identifying the dose-risk associations would improve RT delivery protocols to limit the occurrence of different forms of AF and, if necessary, initiate appropriate treatment.

**Trial Registration:**

ClinicalTrials.gov NCT06073509; clinicaltrials.gov/study/NCT06073509?id=NCT06073509&rank=1.

**International Registered Report Identifier (IRRID):**

DERR1-10.2196/67875

## Introduction

### Background

Breast cancer (BC) is the most common cancer in women [1]. Early screening and advances in treatment, including surgery, chemotherapy, hormone therapy, and targeted therapies, have significantly improved survival rates. Radiotherapy (RT) plays a crucial role in BC management by reducing the risk of local recurrence and improving overall survival, particularly following breast-conserving surgery [2]. Despite its efficacy, RT for BC can induce adverse effects on the heart, particularly when the heart is near the radiation field. Cardiac complications can include coronary artery disease, pericarditis, valvular heart disease, and myocardial infarction [3-5]. Emerging evidence also indicates a risk of atrial fibrillation (AF) in patients with BC that may manifest months to years after treatment [6,7]. AF is a common cardiac arrhythmia characterized by an irregular and often rapid heart rate [8,9] that can eventually lead to conditions with significant health impacts (stroke, heart failure, dementia) [10-12]. AF screening is challenging, further compounded by its frequent asymptomatic nature, which has often gone undetected in retrospective noninterventional studies. It is therefore crucial to further investigate the risk of AF in patients with BC as it may compromise the quality of life and survival of patients.

### Atrial Fibrillation in Patients With Breast Cancer

Cancer patients have an increased risk for developing AF, varying according to cancer type and stage [7] and medical treatments for cancer [13]. It has been suggested that patients with BC may also not be exempt from AF risk, but results between studies are conflicting. A recent study based on the Medicare database showed that the incidence of AF is significantly higher in patients following the year of the diagnosis of BC compared to a control population without cancer (3.3% vs 1.6%, *P*<.05) [6]. With longer follow-up, several retrospective studies have indicated higher AF rates in patients with BC compared to the noncancer population [14-21], but these results were not confirmed in a Danish cohort [22], a US case-control study [23], and a meta-analysis [19]. The impact of age could explain such discrepancies between studies, as illustrated in a large cohort study using the National Inpatient Sample database in the United States based on 40,030,380 adults’ hospitalization for cancer, where patients with BC aged >80 years had an increased risk of AF [24]. Similar results were observed in a systematic review and meta-analysis that found that older patients (age ≥65 years) with BC have a 4.5 times’ higher incidence of AF than younger patients with BC (8.6% vs 1.9%) [18]. Other important factors could impact the risk of AF in patients with BC, such as the medical treatments for cancer, including thoracic surgery, chemotherapy, endocrine therapy, or RT [25]. The effects of surgery and chemotherapy on AF risk have been widely studied, but few studies have documented the risk of AF after RT.

Indeed, some scarce studies have specifically analyzed the link between AF and RT for BC. In 2 studies, RT was associated with a significantly increased risk of AF when compared to a control population without cancer (relative risk [RR] 1.82, 95% CI 1.07-3.08) [26]; hazard ratio [HR] 1.25, 95% CI 1.05-1.48 [27]). However, in a US cohort study, the risk of AF was not significantly higher in patients treated with RT compared to the general population [28].

### Screening of Atrial Fibrillation: A Challenge

Most of the aforementioned studies were retrospective and based on medical records and hospitalization for identification of AF events and thus presented the limitation of potential undercapture of events, which is a concern for AF, as explained next.

AF is the most common arrhythmia, which affects 1% of the general population and about 10%-20% of individuals aged 80 years or more. The definitive method for diagnosing AF is through visual inspection of an electrocardiogram (ECG). Although an irregular pulse might suggest the possibility of AF, an ECG is essential to confirm the diagnosis. Although persistent AF is easily detected, other forms are hard to diagnose despite symptoms. This is due to the paroxysmal nature of many types of AF that might disappear on the way to the hospital for a 12-lead ECG evaluation. Holter monitoring might fail to detect paroxysmal AF if it does not occur on the day of examination. Moreover, AF may be asymptomatic [29], and the challenge in early screening of AF is compounded by its often asymptomatic nature, as approximately one-third of individuals with this form of arrhythmia are unaware of its presence [30]. This has led to the concept of silent AF, which describes subclinical, asymptomatic episodes of paroxysmal AF.

To overcome these limitations of 12-lead ECG and Holter monitoring, advancements in wearable technology have led to the development of smartwatches with integrated ECGs (30-second single-lead electrocardiogram [SL-ECG] recording), which allows continuous monitoring of cardiac activity and can help identify silent AF episodes that might be missed during a single evaluation [31-33]. The effectiveness and reliability of ECG smartwatches for the screening of AF has been demonstrated in several studies (sensitivity 55%-97.3%, specificity 60%-98.2% [31,34-36]), in particular for silent or paroxysmal AF, by measuring continuous cardiac frequency and SL-ECG [37-39]. Moreover, ECGs generated by smartwatches are considered effective and noninferior to ECG/composite 12-lead ECG/Holter/patch monitoring for AF screening [40,41], even if validation of the diagnosis through manual interpretation by a physician is required [39]. Regarding the adherence rate of smartwatch wearing in individuals over 65 years, the PulseWatch trial showed a decrease after 1 month (baseline: 73%; day 30: 63%; *P*<.05) [42], illustrating the limitations of a long period (>1 month) of follow-up with smartwatches in the frame of an AF-screening campaign.

Recently, the European Heart Rhythm Association published recommendations on the use of digital devices to detect and manage AF [43]. It concluded that systematic screening using intermittent ECGs may be beneficial to detect AF in individuals aged >65 years with comorbidities increasing the risk of stroke. RT has been shown to increase the risk of cardiovascular and cerebrovascular diseases [4,44] in patients with BC. It may thus be important to consider AF screening for patients who have undergone RT for BC, due to their increased risk of cardiovascular complications. The interest and feasibility of opportunistic screening for AF in women treated with RT for BC have never been investigated.

### Relationship Between Cardiac Exposure to Radiation and Atrial Fibrillation Among Patients With Breast Cancer

Women treated with RT for BC present clinical characteristics at baseline that may induce a higher risk of cardiac events independent of irradiation, and it is consequently crucial to consider cardiac dosimetry to better understand the potential association between RT and the occurrence of AF and then establish a causality link. The association between the mean heart radiation dose and AF risk have been poorly investigated. The Northern Ireland Cardiac Health Events After Radiotherapy (NI-HEART) Study of patients with lung cancer found a dose-response relationship with HR=1.75 (95% CI 1.03-2.97) [45]. For BC, a similar association with the mean heart radiation dose was observed, with HR=1.23 (95% CI 1.15-1.32) [46]. However, radiation exposure of the heart during RT is not homogeneous, and the mean heart radiation dose is often not relevant to evaluate cardiac substructure RT doses [47] that may be more relevant to evaluate associations with AF. Some studies have begun to explore the dosimetry of cardiac substructures, and recent data have demonstrated associations between cardiac substructure radiation doses and specific cardiac events or mortality in patients. In a cohort that included 238 patients with esophageal cancer treated with RT, increasing the mean left atrial radiation dose was associated with AF risk (with 30% for every 10 Gray increase) [48]. In the field of arrhythmias and conduction disorders, this was observed for the right atrium and the sinoatrial node and the risk of conduction disorders in patients with BC [49-51]. In the case of AF, the arrhythmogenic tissue is often located in the pulmonary veins (PVs), but these structures are not yet considered organs at risk during RT planning, despite a potential relevant association, as suggested in the NI-HEART Study, which investigated the PV radiation dose and the risk of AF in patients with lung cancer following RT. Dose metrics for both the left (V55) and the right (V10) PVs were associated with the incidence of new AF (HR 1.02, 95% CI 1.00-1.03, *P*=.005, and HR 1.01, 95% CI 1.00-1.02, *P*=.033, respectively) [52]. Further research is needed to accurately assess the radiation doses absorbed by some of these substructures and to determine whether the exposure of certain potentially critical structures can better predict the risk of cardiac arrhythmias, particularly AF, in patients with BC. Indeed, identifying critical substructures for AF in women undergoing RT for BC and demonstrating the dose-risk relationship could thus improve RT delivery protocols to limit the occurrence of these arrhythmias.

Manual contouring in radiation treatment planning is time-consuming, requiring meticulous effort to ensure precise delineation of volumes. In recent years, artificial intelligence (AI), particularly deep learning–based autosegmentation models, has emerged to mitigate this workload. These models are used for contouring organs at risk, offering significant improvements in consistency and efficiency, and substantially reducing the time required [53]. However, such deep learning autosegmentation models are scarce or inexistant for specific cardiac substructures, such as conduction nodes [54] or PVs [55], and remain to be developed.

### Study Rationale

Studies on AF and its potential link with RT for BC are relatively limited and have several limitations: the probable undercapture of AF events, particularly in the case of silent AF; the lack of details on the identification or diagnosis of AF; and the lack of precise dose metrics data for cardiac substructures.

In the population of patients with BC treated with RT, asymptomatic forms of AF are often missed in retrospective data collection, which typically identifies only symptomatic cases. Our study hypothesizes that at the time of the last follow-up visit (ie, 5 years post–breast RT by an oncologist), an opportunistic single-timepoint screening combining the use of a smartwatch and a cardiological evaluation, including an ECG and an echocardiogram, could help identify these asymptomatic cases. This would facilitate the management and potential treatment of these patients and increase the completeness of identifying incident cases potentially associated with RT.

Cardiac irradiation related to RT for BC can lead to an increased risk of cardiovascular diseases a few months to several years after treatment. However, precise data for AF, to establish a potential dose-response relationship, are sparse. Our study hypothesizes that the risk of AF could be associated with the radiation dose absorbed by critical cardiac substructures.

### Objectives

In this context and to explore these hypotheses, we will implement the Watch Your Heart After Radiotherapy for Breast Cancer (WATCH) cohort study combining a retrospective design and cross-sectional screening, which aims to investigate AF, including asymptomatic forms, that has occurred in patients with BC during 5 years following RT.

The *primary objective* of the study is to assess the incidence of symptomatic or asymptomatic AF occurring throughout a 5-year follow-up after RT and to identify nonradiation and radiation risk factors for the occurrence of such events.

The *secondary objectives* are as follows:

- Perform a dosimetry evaluation of radiation doses absorbed in the heart and cardiac substructures (chambers, conduction nodes, coronary arteries, PVs) based on autosegmentation models developed with deep learning algorithms.
- Investigate whether the risk of AF is associated with cardiac irradiation characterized by these absorbed radiation doses.
- Assess the incidence of other arrythmias or cardiac diseases throughout the 5-year follow-up after RT and analyze these risks according to the level of cardiac irradiation.
- Evaluate the satisfaction and usability of the smartwatch with an integrated ECG in patients with BC.

## Methods

### Study Design

**Figure 1:**
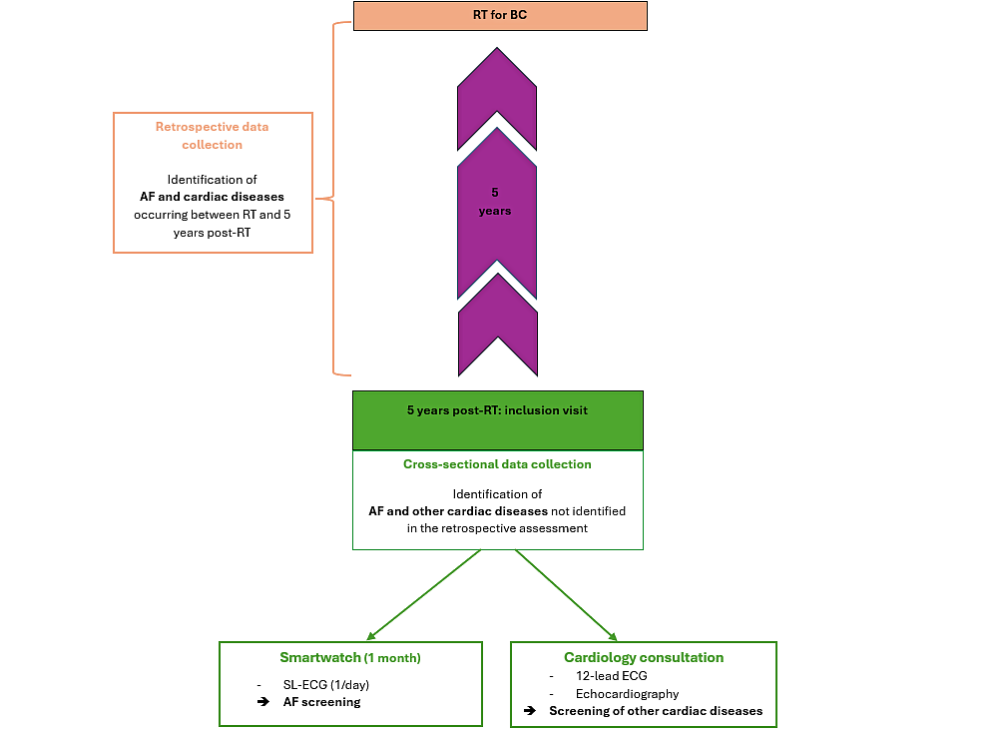
WATCH study design. AF: atrial fibrillation; BC: breast cancer; ECG: electrocardiogram; RT: radiotherapy; SL-ECG: single-lead electrocardiogram; WATCH: Watch Your Heart After Radiotherapy for Breast Cancer.

WATCH is a cohort study implemented as a retrospective and cross-sectional study [56] that will include female patients with left- and right-sided BC treated with postoperative RT after primary breast-conserving surgery. A cross-sectional collection at least 5 years after RT at the time of the last follow-up visit with the oncologist will be performed through smartwatch screening and a cardiology consultation (ECG and echocardiography) for AF screening. This 5-year post-RT timepoint for cross-sectional analysis has been chosen for 2 main reasons: first, to catch an opportunity of the last follow-up consultation with the oncologist to present the study to the patients and, second, a delay of 5 years post-RT appeared relevant to detect AF in patients with BC treated with RT, as observed in our previous work [49]. The retrospective collection of information will be based on questionnaires delivered to the patients to complete and checked against medical records. Figure 1 shows the study design.

The study was registered with ClinicalTrials (NCT06073509) on September 10, 2023.

### Ethics Approval

This study will be conducted in accordance with the Declaration of Helsinki (amended at the 64th World Medical Association General Assembly, Fortaleza, Brazil, October 2013) and the principles of Good Clinical Practice and the Medical Research Involving Human Subjects Act (WMO). This project aims to evaluate a medical device or a device used for nonmedical purposes and is framed by Regulation (EU) 2017/745 on medical devices (MDR). This study received ethical approval from the French Southwest Committee for Protection of Persons and from the National Agency for Medical and Health Products Safety (IDRCB number 2023-A01622-43). Participants enrolled in the study will provide written informed consent. To ensure privacy and confidentiality, all data collected in this study are de-identified prior to analysis, with no personally identifiable information retained.

### Study Population

In this study, we plan to include 200 female patients with unilateral BC, aged over 65 years old at inclusion and treated at least 5 years ago with postoperative modern photon-based RT after surgery. For this study, we will focus on patients aged >65 years, as recommended by the European Heart Rhythm Association [43], which concluded that systematic screening using intermittent ECG may be beneficial to detect AF in individuals aged >65 years with comorbidities increasing the risk of stroke. Patients with a history of cancer or AF before RT or with recurrent cancer (BC or other) after RT will be excluded. Inclusion and exclusion criteria are summarized in Textbox 1.

**Inclusion and exclusion criteria.**

**Inclusion criteria:**

- Women surgically treated for left or right breast cancer (BC) and for whom adjuvant treatment is radiotherapy (RT), with irradiation of the breast or chest wall irradiation and possibly ganglion chains
- Adjuvant RT performed between 5 years before enrollment
- A 5-year post-RT follow-up oncologist consultation performed in one of the investigating centers
- Age 65 years or more at enrollment
- Owning a smartphone and able to understand and use digital tools alone or with the help of a caregiver
- Consented to a connected follow-up
- Affiliated with a social security system or equivalent
- Volunteering to participate in the study and providing signed informed consent

**Exclusion criteria:**

- History of cancer before RT for BC
- History of atrial fibrillation (AF) before RT for BC
- Recurrent cancer (BC or other) after RT for BC

### Recruitment and Study Procedures

Patients will be enrolled at the Clinique Pasteur in Toulouse and at the François Baclesse Center for Cancer Control/Caen University Hospital in Normandy. Until now, enrollment has begun only at the Clinique Pasteur.

#### Before Inclusion

Potential participants undergo prescreening by the oncologist during their final post-RT follow-up consultation (approximately 5 years after RT) to ensure eligibility. This is performed before any study procedure is conducted. The participants are enrolled formally only after they sign the informed consent form at the inclusion visit.

#### Inclusion Visit

Participants complete a medical questionnaire to provide information about their medical history prior to RT and all treatments and cardiovascular diseases that may have occurred since the end of RT (see details in the *Data Collection* section). The participants then receive the *Withings ScanWatch* (Withings SA) smartwatch, and they install the Withings HeathMate app on their smartphone to continuously record their cardiac activity and register ECGs. The watches are purchased by the Authority for Nuclear Safety and Radiation Protection (ASNR), the study sponsor. The choice of Withings ScanWatch was based on several reasons. Many smartwatches integrated with ECG recording currently meet the European Conformity (CE) standards. A recently published study [31] concluded that all evaluated devices have high diagnostic accuracy. An inclusion criterion for the WATCH Study specifies that patients must own a smartphone, with no restrictions on the smartphone brand. An analysis of the user manuals of the different watches led us to choose Withings ScanWatch, as it is the only one compatible with all smartphone brands. Moreover, feedback from the cardiologists involved in the study was positive regarding this smartwatch: accurate ECG recordings, quite simple for patients to use, and good battery life. It should be noted that there was no conflict of interest among the investigators in the choice of Withings ScanWatch.

The functions of the smartwatch and its application are explained to the participants. They are required to wear the loaned smartwatch for 1 month and record an ECG at least once per day or when the smartwatch automatically detects an abnormal heart rhythm and suggests that the user record an ECG.

#### Screening Visit

One month after the inclusion visit, all recorded smartwatch-based ECG data are emailed as a PDF file by the patients to the study cardiologists. To prevent data collection failure due to elderly adults’ unfamiliarity with smartphone usage, especially when sending an email with a PDF file attached, practice time is scheduled during the screening visit with clinical researchers (practice for 1 hour). During this visit, if necessary, patients can be accompanied by their caregivers, who will be able to transfer the PDF files. The patients complete a questionnaire to evaluate their satisfaction with and the usability of the smartwatch integrated with ECG recording, reset the smartwatch, and return it. The smartwatch is then loaned to another patient enrolled in the study. Finally, a cardiology consultation including a 12-lead ECG completed with transthoracic echocardiography (TTE), providing a comprehensive assessment of cardiac structures and functions, is performed (see details in the *Data Collection* section). Criteria for diagnosing asymptomatic AF and AF using instruments and auxiliary examinations in this study will follow the most recent guidelines for AF management [57].

### Data Collection

#### Cancer, Treatment, and Medical History Before and After RT

Information about cancer and its treatment (surgery, chemotherapy, RT, hormonotherapy) are collected through hospital medical records. The retrospective collection of information, which aims to identify cardiovascular diseases prior to RT and those that occurred between RT and 5 years post-RT, is based on medical questionnaires filled by the patients and checked against their medical records (see Textbox S1 in Multimedia Appendix 1).

#### Cardiac Examinations

The cross-sectional data collection, which aims to identify AF and other cardiac diseases at 5 years post-RT, not previously identified in the retrospective data collection, is based on screening for cardiac diseases. This screening is conducted by recording data from the Withings ScanWatch smartwatch, confirmed by an ECG, and validated by a cardiologist. In addition to AF screening, a 12-lead ECG and a TTE are performed (see Textbox S2 in Multimedia Appendix 1). Patient satisfaction with and usability of the smartwatch are assessed using a satisfaction survey [58] and the System Usability Scale (SUS) score [59] (see Textbox S3 in Multimedia Appendix 1).

#### Cardiac Dosimetry

For cardiac dosimetry, autosegmentation algorithms are being developed based on AI/deep learning to assess the radiation dose to potentially critical substructures for the occurrence of AF (whole heart, cardiac chambers, sinoatrial node, atrioventricular node, coronary arteries, PVs). These algorithms will use convolutional neural networks trained on large datasets of annotated computed tomography (CT) images to identify and contour cardiac substructures with high precision. Specifically, the model used in this study will be trained and validated using a dataset of CT images annotated by expert radiation oncologists and cardiologists to ensure anatomical accuracy and reproducibility [47,50]. Validation metrics, such as the Dice similarity coefficient (DSC), the Hausdorff distance, and the mean surface distance, will be used to evaluate the model’s performance against expert contours.

For each patient included in the WATCH cohort, CT images are collected with Digital Imaging and Communications in Medicine (DICOM) data. These CT images will be contoured with our deep learning segmentation tool to provide mean radiation doses and dose-volume histograms for all critical cardiac substructures. The application of AI in this study will allow for reproducible, time-efficient, and standardized dosimetry, addressing interobserver variability associated with manual contouring. Furthermore, the algorithms will be tested for robustness across different scanner models and imaging protocols to ensure generalizability. Nevertheless, manual review and correction by experienced clinicians will be performed for a subset of cases to further verify segmentation accuracy and consistency, aligning with the best practices for incorporating AI in clinical research.

### Outcome Measurement

#### Primary Outcome

The difference in the occurrence of AF (questionnaire + SL-ECG based) throughout the 5-year follow-up after RT according to heart exposure (moderate or low exposure: <75th percentile of radiation dose distribution for the whole heart; and high exposure: ≥75th percentile of radiation dose distribution) will be measured as the primary outcome of interest for this study.

#### Secondary Outcomes

As mentioned before, the secondary outcomes are:

- Cardiac substructure dosimetry (cardiac chambers, conduction nodes, coronary arteries, and PVs)
- Association between AF occurrence and cardiac substructure dosimetry
- Occurrence of other arrythmias and conduction disorders (12-lead ECG based) throughout the 5-year follow-up after RT
- Occurrence of other cardiac dysfunction, cardiomyopathies, and valvular heart diseases (TTE based) throughout the 5-year follow-up after RT
- Association between other arrythmias and conduction disorders or other cardiac diseases, and cardiac substructure dosimetry
- Patient satisfaction score using the Lickert scale and usability score using the SUS

### Sample Size Calculation

The calculation of the required sample size was conducted within the framework of an exploratory study. The required number of patients was set to 200 based on a statistical power of 80% with a type I error rate of 5% and theoretical percentages of AF incidence of 10% for the group of patients considered moderately exposed (<75th percentile of the average heart radiation dose distribution) and 25% for the group of patients considered highly exposed (>75th percentile of the average heart radiation dose distribution) [60]. We plan to include these 200 patients in approximately 30 months.

### Planned Statistical Analysis

We will summarize the baseline data (description of the cancer and treatments, history of cardiovascular diseases, cardiac dosimetry) using descriptive statistics: means (SDs) will be used for continuous data with a normal distribution, medians (IQRs) for skewed data, and percentages for categorical data. Continuous data will be compared between cases and noncases of AF using the *t* test or the Wilcoxon Mann-Whitney test, and categorical variables will be compared using the chi-square test. All tests will be bilateral, with α=5%.

The estimation of the incidence of AF and other cardiovascular pathologies occurring in the 5 years following RT will be carried out based on data collected during the cardiology consultation (SL-ECG screening, 12-lead ECG, and echocardiography) and the medical questionnaire. A univariate analysis using regression models will identify the risk factors associated with the studied cardiovascular pathologies, other than cardiac radiation exposure (ie, individual and medical characteristics). Subsequently, only variables with *P*<.20 will be retained for multivariable analyses [61]. Estimations of the risk of AF and other cardiovascular diseases according to cardiac radiation doses will be performed using regression models adjusted for the risk factors identified in the univariate analysis. To limit issues in the multivariate analysis due to correlation between dosimetry parameters, each dosimetry parameter will be analyzed separately. Analysis will be performed using the data analysis and statistical software SAS version 9.4.

## Results

The study was funded in October 2023 and is still in progress. Enrollment and data collection began in October 2023, and we aim to cover 200 patients by mid-2026. A second inclusion center is planned to be opened by mid-2025. Data analysis will start in June 2026, and the results are expected by the end of 2026.

## Discussion

### Summary

This study protocol aims to evaluate the risk of AF in patients with BC who have undergone RT, addressing a critical gap in understanding cardiovascular complications of BC treatment. AF is a common arrhythmia with potential to cause severe complications, and its risk may be heightened by the incidental cardiac radiation exposure during RT for BC. Given the increasing survival rates in patients with BC, assessing long-term cardiovascular outcomes has become essential for optimizing post-treatment care.

Several studies have investigated the association between RT for BC and AF [6,26,62]; however, these studies have not provided precise dosimetry data for cardiac substructures, and it remains unclear whether this association is causative or whether cancer and AF just share the same pathophysiologic mechanisms. Our study aims to enhance our understanding of how radiation dose distribution across specific cardiac regions correlates with AF incidence. Our study’s approach involves detailed dosimetry analysis of cardiac substructures using deep learning–based autosegmentation, including the left and right atria, the left and right ventricles, coronary arteries, the conduction system, and PVs. This precision in quantifying radiation doses received by these areas will allow for a more granular assessment of radiation exposure and its impact on AF risk, distinguishing our study from studies that have evaluated whole-heart doses [26]. Univariate and multivariable regression analyses will be used to isolate the effects of these localized radiation doses, while adjusting for confounding factors, such as age, comorbidities, and chemotherapy. This will provide an understanding of the dose-response association and help identify high-risk cardiac regions.

With a retrospective design, previous studies that have investigated AF occurrence in patients with BC may have undercaptured AF events, particularly in the case of silent AF. Traditional methods, such as standard ECGs and echocardiography, have proven efficacy in detecting AF but often lack continuous monitoring capability essential for capturing transient AF episodes. Smartwatches, equipped with SL-ECG recording, offer a promising adjunct, providing continuous real-time data on heart rhythm. To investigate AF incidence over 5 years post-RT, our study combines retrospective patient-reported outcomes and cross-sectional screening based on smartwatches, in addition to traditional diagnostic methods (ECG and echocardiography). Patient questionnaires capture symptoms and medical history, offering a broader context for interpreting cardiovascular health and identifying potential noncardiac factors influencing AF risk. ECG and echocardiography will provide objective data on cardiac rhythm disturbances and structural and functional cardiac abnormalities. More particularly, in patients with AF, echocardiography will allow us to investigate the underlying etiology and assess the risk of complications, while patients’ medical records will provide information about the timing and risk factors for the occurrence of AF and other arrhythmias/conduction disorders during the 5 years following RT. This multimodal approach will allow for a comprehensive understanding of AF development in the context of radiation exposure and patient-specific factors.

### Limitations

However, this study presents several limitations. First, the sample size is relatively small, which, although suitable for an exploratory analysis, will limit the generalizability of our findings. Future studies should incorporate larger cohorts to enhance the statistical power and validity of the results. Due to the inclusion criteria, which selected only patients aged more than 65 years and who own and are able to use multiple digital tools (smartphone, smartwatch, email) or at least be assisted by a caregiver, our estimates of AF at the scale of our population will not be applicable to all patients with BC. However, results on the association between cardiac radiation exposure and AF will not be biased by such potential selection bias. Moreover, this exploratory study does not intend to provide exhaustive figures of AF in all patients with BC who are >65 years old, but it will compare AF between high- and low-cardiac-exposure groups. Selection bias, if any, will not be impacted by the cardiac radiation exposure level of patients. Thus, our study should not provide biased results regarding the potential association between cardiac radiation exposure and AF risk. Second, the study population is limited to older adults, potentially overlooking age-related variability in radiation-induced AF risk. Expanding the research to include a broader age range will provide a more comprehensive understanding of the age-dependent effects. Third, for retrospective collection of medical data, some patients may provide incomplete information, resulting in missing data. However, this issue will be limited by additional collection of missing data based on medical records. Last, the follow-up period is limited to 5 years and AF screening performed 5 years after RT. Missed screening of AF should be limited by a combination of retrospective and cross-sectional design. The retrospective part will allow us to collect data on the occurrence of AF based on patients’ questionnaires and medical records for a duration of 5 years post-RT. In cross-sectional screening 5 years after RT, patients will wear their smartwatch for a duration of 1 month. This duration will not avoid missed screening for patients with AF that would not occur during this period. Moreover, this duration and opportunistic single-timepoint screening may not fully capture the long-term incidence of AF or the shorter effects post-RT, suggesting the need for both shorter-term and extended follow-up in future investigations to better understand the timing and progression of AF posttreatment.

### Conclusion

To conclude, this study will refine our understanding of radiation-induced AF after RT, a topic almost absent from the European Society of Cardiology (ESC) guidelines on cardio-oncology [63]. Given the relatively high incidence rate of AF from a certain age in the general population, identifying increased AF risk linked to RT could result in a significant number of RT-attributable cases. Detecting silent AF may necessitate medical/preventive management for certain patients to limit the risk of stroke. The use of smartwatches has never been studied in a specific population, such as patients treated with RT for BC. This study will evaluate the interest of patients and cardiologists in this type of connected tool within this population. The results of this project could provide recommendations for primary and secondary prevention strategies to limit RT sequelae and improve patients’ quality of life.

## Appendix

Multimedia Appendix 1 Medical questionnaire: list of items; cardiology consultation: list of items; and questionnaire to evaluate patient satisfaction with and usability of the smartwatch integrated with electrocardiogram (ECG) recording.

## Abbreviations

AF: atrial fibrillation
AI: artificial intelligence
BC: breast cancer
CT: computed tomography
ECG: electrocardiogram
HR: hazard ratio
NI-HEART: Northern Ireland Cardiac Health Events After Radiotherapy
PV: pulmonary vein
PV: pulmonary vein
RT: radiotherapy
SL-ECG: single-lead electrocardiogram
SUS: System Usability Scale
TTE: transthoracic echocardiography
WATCH: Watch Your Heart After Radiotherapy for Breast Cancer

## Data Availability

The datasets generated and analyzed during this study are available from the corresponding author upon reasonable request.
