# Supplementary material for: Screening and Risk Analysis of Atrial Fibrillation After Radiotherapy for Breast Cancer: Protocol for the Cross-Sectional Cohort Study “Watch Your Heart (WATCH)”
Source: JMIR Res Protoc. 2025 Jun 4;14:e67875. doi: 10.2196/67875 (PMC12177427; doi:10.2196/67875)
Supplement: Multimedia Appendix 1 [file resprot_v14i1e67875_app1.docx]

**Textbox S1.** Medical questionnaire: list of items.

| **Provide an answer for each item: yes/no; if yes, provide the date.** | |
| --- | --- |
| **Cardiovascular conditions** | **Respiratory conditions** |
| - Congenital heart malformation | - Chronic obstructive pulmonary disease |
| - pulmonary hypertension | - Pulmonary embolism |
| - Carotid stenosis |  |
| - Transient ischemic attack | **Endocrine conditions** |
| - Stroke | - Hyperthyroidism |
| - Intermittent claudication | - Hypothyroidism |
| - Atherosclerosis | - Thyroidectomy |
| - Aortic aneurysm | - Metabolic syndrome |
| - Deep vein thrombosis |  |
| - Family history of myocardial infarction |  |
| **Cardiac diseases** |  |
| - Acute myocardial infarction | - Cardiomyopathy |
| - Recurrent myocardial infarction | - Endocarditis |
| - Coronary angioplasty +/- stent | - Pericarditis |
| - Coronary artery bypass | - Myocarditis |
| - Angina pectoris | - Valvular heart disease |
| - Heart failure |  |
| **Cardiovascular treatments:** type, date of start of treatment | |
| **Cardiac arrhythmias** |  |
| - Sinus node dysfunction | - Polymorphic VT/torsades de pointes |
| - First-degree atrioventricular (AV) block | - Syncope with inducible VT or VF |
| - Second-degree AV block | - Prophylactic (undocumented) |
| - Complete AV block | - Supraventricular tachycardia |
| - Left bundle branch block | - Wolff-Parkinson-White syndrome |
| - Right bundle branch block | - Atrial fibrillation |
| - Permanent atrial fibrillation and AV block | - Atrial flutter |
| - Ventricular fibrillation (VF) | - Atrial tachycardias |
| - Ventricular tachycardia (VT) | - Cardiac arrhythmia, unspecified |
|  |  |
| **Treatment for cardiac arrhythmia** |  |
| - Antiarrhythmic treatments (amiodarone, flecainide, propafenone, sotalol, beta-blockers, verapamil, digoxin) | |
| - Anticoagulant treatments: VKAs (fluindione, acenocoumarol, warfarine), DOACs (dabigatran, rivaroxaban, apixaban) | |
| - Pacemaker implantation |  |
| - Defibrillator implantation |  |
| - Resynchronization |  |
| - Ablative treatment: ablations of flutter, AF, atrial tachycardia, AVNRT, accessory pathway, VT | |

**Textbox S2.** Cardiology consultation: list of items.

| **Provide an answer for each item: yes/no; if yes, provide the date.** |
| --- |
| - Age, weight, height - Blood pressure - Hypertension - Smoking status - Dyspnea (NYHA) - Diabetes - Cardiovascular treatments - Noncardiovascular treatments - Fasting blood glucose, urea, creatinine, eGFR - LDL cholesterol, HDL cholesterol, total cholesterol, hypercholesterolemia, triglycerides - CHA2 DS2 VASc score - HAS-BLED score |
| **Smartwatch ECG**   - Number of abnormal heart rhythm alerts - Number of recorded ECGs - Number of suspected episode(s) of AF - Analysis of smartwatch ECG tracings, comments |
| **12-lead ECG**   - Sinus rhythm: yes/no - Atrial fibrillation: yes/no - Other rhythm (please specify) - PR interval duration: …… msec - AV block: yes/no; if yes, please specify: 1st degree AV block / 2nd degree AV block Mobitz type 1 / 2nd degree AV block Mobitz type 2 / 3rd degree AV block - QRS duration - Bundle branch block: yes / no; if yes, please specify: right bundle branch block / left bundle branch block / other - QRS axis - Premature atrial contractions (PAC): yes / no; if yes, specify the number. - Premature ventricular contractions (PVC): yes / no; if yes, specify the number. - Myocardial infarction sequelae: yes / no; if yes, specify the territory. - Normal ECG: yes / no |
| **Transthoracic echocardiography**  **Echogenicity:**  **Aortic root:**   - Sinuses: …… mm - Sinotubular junction: …… mm - Segment I: …… mm   **Pulmonary valve:** normal, physiological microregurgitation  **Left ventricle:**   - IVSd: …… mm; LVEDD: …… mm; LVESD: …… mm; LVPWd: …… mm - LVEF: …… % by biplane Simpson - Global longitudinal strain …… % - Indexed end-systolic volume: …… mL/m² - Indexed end-diastolic volume: …… mL/m² - Normal segmental wall motion   **Aortic valve:**   - Tricuspid - LVOT diameter: …… mm; LVOT VTI: …… cm - Ao VTI: …… cm - Mean gradient: …… mmHg; VMax: …… m/s; velocity ratio: …… - Aortic valve area: …… cm² - No regurgitation   **Mitral valve:**   - Normal, no regurgitation, no stenotic effect - Mean gradient: ……. mmHg; mitral valve area: ……   **Left atrium:** …… mL/m²   - Reserve strain: ……% - Systolic strain: ……%   **Right atrium:** …… cm²  **Right ventricle:**   - RVEDA: …… mm²; RVESA: …… mm²; FAC: …… % - TAPSE: …… mm; S' wave: …… cm/s; free wall strain (3 segments): …… % - No RVH   **Tricuspid valve:** normal, physiological microregurgitation  **Pulmonary pressures:**   - Pulmonary acceleration time: …… ms - sPAP: …… mmHg - mPAP: …… mmHg - dPAP: …… mmHg   **Volumetric parameters:**   - E/A = ……; E/E'lat = ……; S/D = …… - IVC: ……   **Pericardium dry** …… |

**Textbox S3.** Questionnaire to evaluate patient satisfaction with and usability of the smartwatch integrated with ECG recording.

| **For each question, use the 5-level scale below to answer:**   \| 1. Strongly disagree \| 2. Disagree \| 3. Neither agree nor disagree \| 4. Agree \| 5. Strongly agree \| \| --- \| --- \| --- \| --- \| --- \| |
| --- | --- | --- | --- | --- | --- |
| **First set of questions for satisfaction (Lickert scale):**   - I understand the benefits of using this smartwatch. - The smartwatch meets my health and clinical monitoring needs. - The smartwatch is easy to use and understand. - The services offered by the smartwatch are useful. - I did not encounter any malfunctions, unavailability, connection issues, or errors while using the smartwatch. - I find that the smartwatch integrates well into my medical monitoring and does not disrupt my current medical care. - The smartwatch improves my current medical care. - The smartwatch does not require additional time for my current medical care and can potentially also save me time. |
| **Second set of questions for usability (System Usability Scale [SUS]):**   - I think that I would like to use this system frequently. - I found the system unnecessarily complex. - I thought the system was easy to use. - I think that I would need the support of a technical person to be able to use this system. - I found the various functions in this system were well integrated. - I thought there was too much inconsistency in this system. - I would imagine that most people would learn to use this system quickly. - I found the system cumbersome to use. - I felt confident using the system. - I needed to learn a lot of things before I could get going with this system. |
